# Study on the treatment of methyl orange contaminated water by activated carbon fiber/copper oxide as persulfate activator under microwave irradiation

**DOI:** 10.1186/s13065-026-01740-1

**Published:** 2026-02-03

**Authors:** Wei Peng, Jiani Li, Hanbing Zhang, Kai Zhang, Zhaoxia Ding

**Affiliations:** PLA Joint Logistics Support Force University of Engineering, Chongqing, 401331 P. R. China

**Keywords:** Advanced catalytic oxidation technology, ACF/CuO nanocomposite, Microwave, Methyl orange

## Abstract

To effectively treat refractory azo dye wastewater, microwave advanced catalytic oxidation technology was adopted to degrade the model pollutant methyl orange using activated carbon fiber (ACF)/CuO as the catalyst and potassium persulfate (K_2_S_2_O_8_) as the oxidant. The optimized experimental parameters and the degradation pathway of methyl orange were determined. The results showed that when the microwave power was 500 W, the irradiation time was 2 min, the dosage of potassium persulfate was 0.6 g/L, and the dosage of ACF/CuO was 10 g/L, the removal rate of methyl orange solution was close to 100%, the COD removal rate was 89.65%, and the TOC removal rate was 72.36%. Mechanism analysis indicated that the double bond was broken to generate acid and *p*-nitrophenol, which were gradually degraded to benzene and phenol under the oxidation of sulfate radical. Subsequently, the benzene and phenol underwent chain cleavage to form maleic anhydride, and part of the benzene, phenol, and the generated maleic anhydride were ultimately degraded to water and carbon dioxide.

## Article Highlights

A combinative strategy involving microwave and heterogeneous catalysis based on ACF/CuO/K_2_S_2_O_8_ was innovatively applied to remove methyl orange contaminated water.

Methyl orange, COD and TOC removal efficiency in the combinative process were achieved to 100%, 89.65% and 72.36%, respectively.

Double bonds in methyl orange were broken to generate acid and *p*-nitrophenol, and they gradually degraded into benzene and phenol by oxidation of sulfate radicals. Water and carbon dioxide are the final degradation products.

## Introduction

With the rapid development of the textile printing and dyeing industry azo dyes such as methyl orange (MO) pose a serious threat to aquatic ecosystems because of their complex aromatic structures and high chemical stability which makes them difficult to remove effectively through traditional biodegradation methods [20]. The treatment of dye wastewater faces two primary challenges: high chromaticity and high organic concentration. To address the environmental pollution caused by organic dyes various methods and technologies have been employed to treat wastewater contaminated with organic dyes. The primary goal is to separate and remove the color-forming substances and to break down these substances to achieve decolorization and degradation of the organic matter [1, 8, 16]. In recent years advanced oxidation processes based on sulfate radicals (SR-AOPs) have become a research hotspot for degrading recalcitrant organic pollutants due to their higher redox potential (2.5–3.1 V) and broader pH applicability. Among various potassium persulfate activation strategies transition metal oxides are widely used owing to their low cost and high efficiency and copper oxide (CuO) demonstrates excellent catalytic activity [18]. However, nanoscale metal oxides are prone to agglomeration during reactions and are difficult to recover, which limits their practical application potential.

To address these limitations heterogeneous catalysts constructed by loading metal oxides onto porous carbon materials have emerged as a viable strategy. Activated Carbon Fiber (ACF) with its large specific surface area abundant microporous structure and functionalized surface serves not only as an ideal support for CuO to enhance the dispersion of active sites but also enriches pollutants through an “adsorption-catalysis” synergistic mechanism thereby shortening the free-radical transport distances [17]. For instance recent studies demonstrate that the strong interaction between surface defects and loaded metal species in carbon-based materials can significantly facilitate electron transfer and accelerate the activation process of potassium persulfate [22].

Furthermore the introduction of external field energy assistance in catalytic oxidation systems has been shown to significantly enhance reaction kinetics. Microwave (MW) irradiation as an efficient heating method not only enables rapid overall heating via dipole polarization and ion conduction but also creates localized “hot spot” effects on the surface of microwave-absorbing materials (e.g. ACF) [9]. This unique non-thermal effect can reduce the reaction activation energy induce the mobility of CuO lattice oxygen on ACF surfaces and improve the mass transfer efficiency at the solid-liquid interface. Recent studies indicate that the coupling technology of microwave with carbon-supported metal catalysts demonstrates significant synergistic effects in degrading organic pollutants with degradation rates far surpassing those of pure thermal activation or conventional heterogeneous catalytic systems [10]. Although microwave-assisted carbon-based catalysts show great potential for potassium persulfate degradation in water treatment, the microscopic mechanisms of CuO/ACF composite materials activating potassium persulfate for methyl orange degradation under microwave irradiation, as well as the evolution of active species, demand further in-depth investigation.

As the most commonly used microwave-induced catalysts activated carbon fiber and copper oxide enhance the treatment effect of microwave advanced oxidation technology on methyl orange dye. They also reduce reaction energy consumption and costs. However both have their respective drawbacks. A high dosage of activated carbon fibers can lead to a minor amount of fibers or ash floating on the water surface after the fibers treated by multiple wastewater soakings are immersed. This can cause a certain level of contamination to the water body. Copper oxide nanowires are difficult to collect and are not practical for future applications. In practical use some of the copper oxide dissolves into the solution resulting in secondary water pollution [13, 19]. Furthermore, when comparing energy consumption and treatment efficacy, both activated carbon fiber and copper oxide exhibit certain levels of consumption. This renders them unsuitable for future practical applications. However, employing a composite material of copper oxide nanowires on activated carbon fiber (ACF/CuO) could reduce catalyst consumption and prevent secondary water pollution, all while maintaining catalytic effectiveness. Additionally, exploring the combined effect of this composite material with microwave advanced oxidation technology could lead to the production of sulfate radicals, which are effective in treating methyl orange dye wastewater.

In this paper, methyl orange dye wastewater was used as the target pollutant. Potassium persulfate served as the oxidant, and ACF/CuO was employed as the catalyst. The study investigated the effects of the microwave-potassium persulfate-ACF/CuO system, optimized its process parameters, and analyzed the catalytic mechanism of ACF/CuO nanocomposites. Additionally, the degradation process of methyl orange was examined, providing a foundation for the practical application of the microwave-potassium persulfate-ACF/CuO system in treating dye wastewater.

## Materials and methods

### Materials and equipment

#### Chemical reagents

Methyl orange, potassium persulfate (K_2_S_2_O_8_), Oxone (KHSO_5_) copper chloride polyethylene glycol 20000 polyvinylpyrrolidone (PVP) polyvinyl alcohol ammonia sodium hydroxide anhydrous ethanol and all other reagents used in the experiment were of analytical reagent (AR) grade sourced from Sinopharm Chemical Reagent Co. Ltd. Meanwhile activated carbon fiber was purchased from Sinopharm Group Chemical Reagents Co. Ltd. and CuO was synthesized using copper chloride and sodium hydroxide [2].

#### Experimental equipment

The ultraviolet-visible photometer T6 (manufactured by Beijing Pulse Analytical General Instrument Co., Ltd.), the ultrasonic reactor SK7210LHC (produced by Shanghai Kedao Ultrasonic Instrument Co., Ltd.), the X-ray polycrystal diffractometer (from Rigaku Denki, Japan), the scanning electron microscope SEM515 (from Philips Co., Ltd.), the electronic balance (from Shanghai Jingke Co., Ltd.), and the microwave oven (a modified Midea microwave oven).

#### Experimental installation

**Fig. 1:**
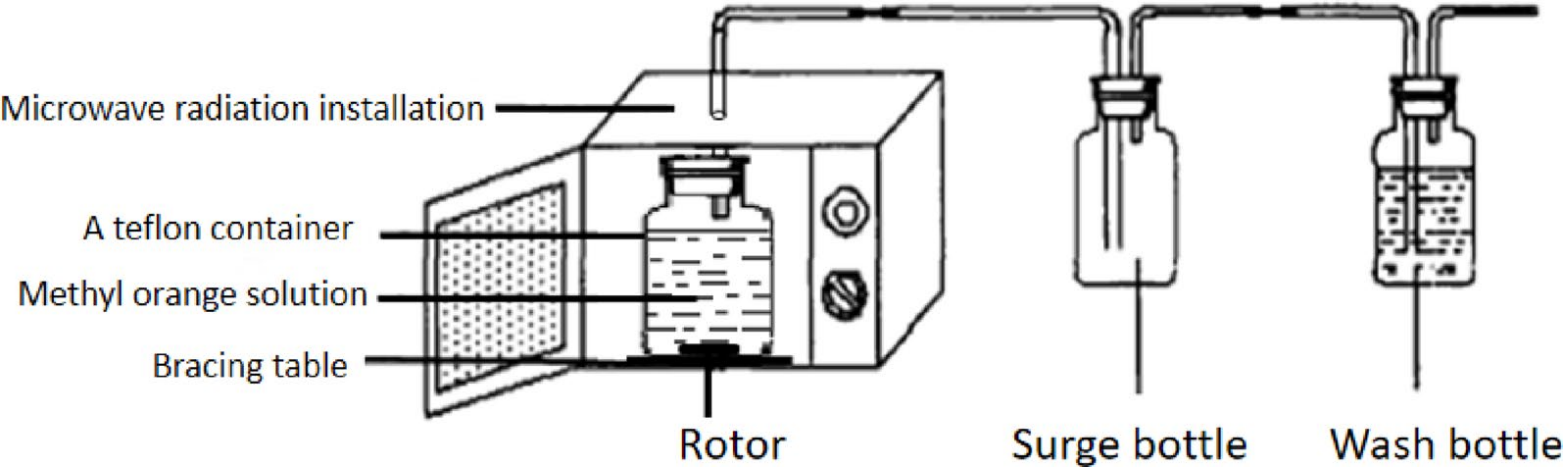
Structure of microwave equipment

The diagram of the modified microwave equipment is shown in Fig. 1. The PTFE reactor was placed on the support table inside the resonance cavity of the microwave oven, ensuring that it avoided the metal guide tube. A hole was drilled at the top of the microwave oven to allow for the insertion of a glass tube, which was sealed with a sealant at the interface to prevent microwave leakage. Buffer bottles and wash bottles were connected to the reactor inside the microwave resonance cavity. The dimensions of the resonance cavity were 340 mm by 320 mm by 220 mm, and the PTFE container had a base diameter of 145 mm and a height of 195 mm. The rotating fan at the top of the microwave oven changed the propagation direction of the microwaves, ensuring that the water samples in the reactor were evenly irradiated.

### Experimental

#### Pre-treatment of activated carbon fiber

The activated carbon fiber was cut into small squares with dimensions of 1 cm by 1 cm and soaked in alcohol for 24 h to remove oil stains from its surface. Then, it underwent ultrasonic cleaning with ultra-pure water several times to wash away any residual alcohol. Subsequently, the activated carbon fiber was placed in ultra-pure water and heated to boiling, and this process was repeated five times to eliminate ash and weak fibers from inside. Finally, the treated fibers were placed in an oven at 60℃ for 12 h.

#### Sensitizing solution preparation

The ratio of stannous chloride to hydrochloric acid was set at 30 g/L to 150 mL/L. The treated activated carbon fibers were immersed in the sensitizing solution for 12 h. Then, they were ultrasonically cleaned with ultra-pure water several times to remove any remaining sensitizing solution. After that, the fibers were placed back in the oven and heated at 60℃ for another 12 h.

#### Activation solution preparation

The ratio of silver nitrate to ammonia was set at 5 g/L to 150 mL/L. The treated activated carbon fibers were immersed in the activation solution for 12 h. Then, they were ultrasonically cleaned with ultra-pure water several times to remove any residual sensitizing solution. Finally, the treated fibers were placed in an oven and heated at 60℃ for 12 h.

#### Activated carbon fiber loaded with copper source

Started with one liter of deionized water, and added 10 g of anhydrous copper sulfate, 10 g of anhydrous sodium carbonate, 15 mL of formaldehyde, 40 mL of a sodium potassium tartrate solution, and 10 mg of 2,2’-bipyridine to it. Introduced the treated activated carbon fiber into the mixture, then adjusted the pH to 12 using hydrochloric acid (HCl) and sodium hydroxide (NaOH) solutions. Ultrasonicated for 10 min, and then performed electrical stirring for 24 h at room temperature. After removing the fibers, washed them several times with ultra-pure water and dried in an oven at 60 °C for 12 h. Subsequently, dried the solution in an oven at 60 °C for an additional 12 h.

#### Preparation of activated carbon fiber loaded with copper oxide (ACF/CuO)

Take out the dried activated carbon fiber loaded with the copper source, placed it in a desiccator, and let the activated carbon fiber cool to room temperature. Then, lay the activated carbon fiber flat to ensure full contact with the oxygen in the air. Subsequently, placed it in an atmosphere furnace at 300 °C, calcine for 2 h, cool it down, and store it in the desiccator.

#### Catalytic experimental methods

The experiment was conducted in a 250 mL special conical flask. The microwave power was adjusted from 0 W to 1000 W, the concentration of the activated carbon fiber loaded copper oxide material was changed from 1 g/L to 20 g/L. 100 mL of a 100 mg/L methyl orange solution was added, and the concentration of potassium persulfate was set from 0 g/L to 1 g/L. The sample was then irradiated for 0 to 3 min to calculate the concentration change and reaction rate of the methyl orange solution.

#### Chemical analysis

An HPLC (Agilent 1260 Infinity II) equipped with a UV detector at a wavelength of 461 nm and a C18 reversed-phase column (150 × 4.6 mm) was employed to detect the concentration of methyl orange. The mobile phase used for HPLC experiments was a mixture of methanol and water (50/50, v/v), which was filtered through 0.22 μm filters before use. The flow rate was set at 1.0 mL/min, and the injection volume was 5 µL. The column temperature was set at 30 °C.

## Results and discussion

### Analysis of micro-structural properties of ACF/CuO

#### Scanning electron microscope (SEM)

**Fig. 2:**
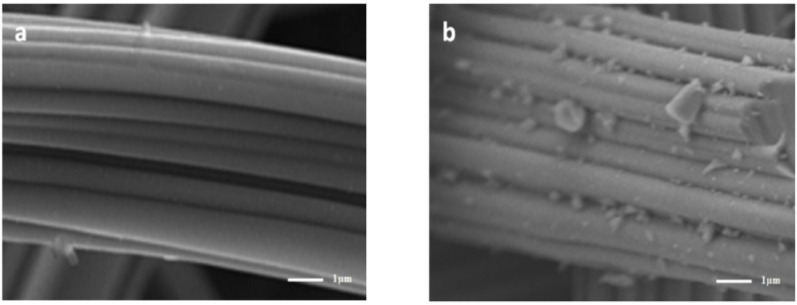
SEM images of (**a**) fiber of carbon, **b** ACF/CuO

To observe the morphology and dimensions of the synthetic products, various morphologies of CuO and ACF/CuO were examined and photographed using scanning electron microscopy. Figure 2 presents the scanning electron microscope images of activated carbon fibers before (Fig. 2a) and after (Fig. 2b) preparation. The surface of the treated activated carbon fiber appears smooth with small pointed copper oxide particles visible on it. There are uniformly distributed CuO nanowires with an average diameter of 40 nm to 60 nm. This is attributed to the copper source deposited on the activated carbon fiber’s surface which when heated to 300 ℃ reacts with the oxygen in the air. The growth of the nuclei is favored at lower temperatures and once the nuclei are formed they grow along the surfactant chains resulting in the formation of copper oxide nanowires [23, 24].

####  Fourier infrared spectra

**Fig. 3:**
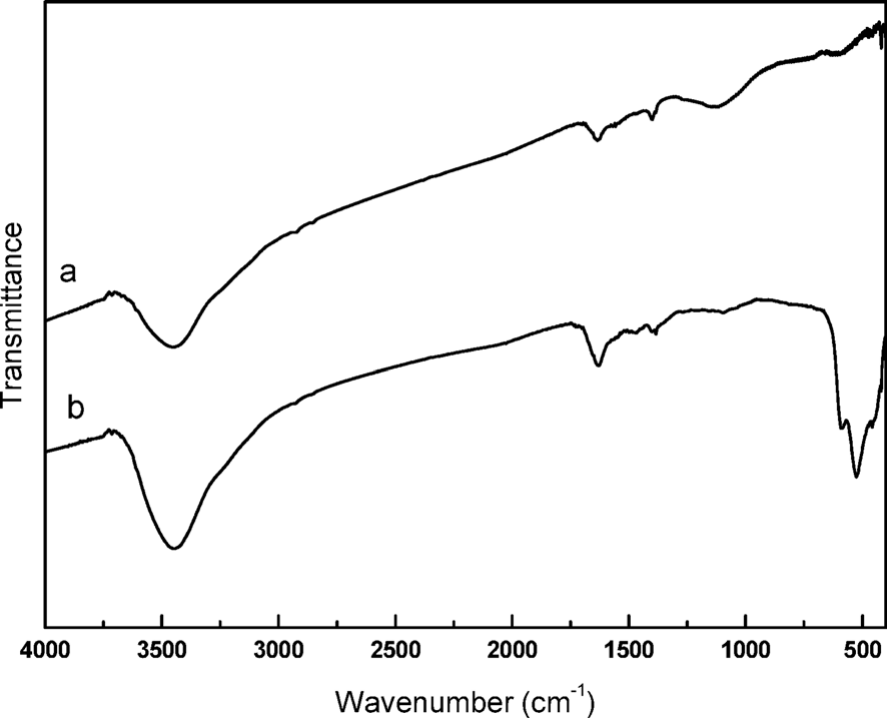
FT-IR images of (**a**) fiber of carbon and (**b**) ACF/CuO

Figure 3 presents the IR spectra of activated carbon fiber and ACF/CuO. Figure 3a depicts the activated carbon fiber, which exhibits a prominent absorption peak at approximately 3444.59 cm^− 1^, primarily attributed to the -OH stretching vibration within the activated carbon fiber. The absorption peak at 1637.62 cm^− 1^ is caused by the vibrations of C = C and C = O bonds in the activated carbon fiber, with the C = C bonds also interacting with those in the benzene ring, resulting in a peak shift to around 1600 cm^− 1^. The absorption peak at 1398.50 cm^− 1^ is due to the stretching vibration of -OH in the water of crystallization, along with the symmetric stretching vibration of -CO2- and the vibrational peaks of C-O in the carboxyl group and phosphate ester. At 1117.01 cm^− 1^, there is a less pronounced absorption peak, which corresponds to the vibrations of C-OH- and C-O-CO.

Figure 3b illustrates the infrared spectra of ACF/CuO. When compared to Fig. 3a, the infrared peaks at 527.17 cm^− 1^, 1637.62 cm^− 1,^ and 3444.59 cm^− 1^ are notably enhanced. This increase is due to the vibrational characteristic absorption peaks of Cu-O at 527.17 cm^− 1^ and the vertical surface dangling bonds in the copper oxide crystals (on the surface of the lattice) at 1637.62 cm^− 1^. Additionally, the telescopic vibrational peak at 3444.59 cm^− 1^is associated with the end-terminated bond [2, 5]. This indicates that copper oxide nanowires have been successfully loaded onto activated carbon fibers.

####  XRD

**Fig. 4:**
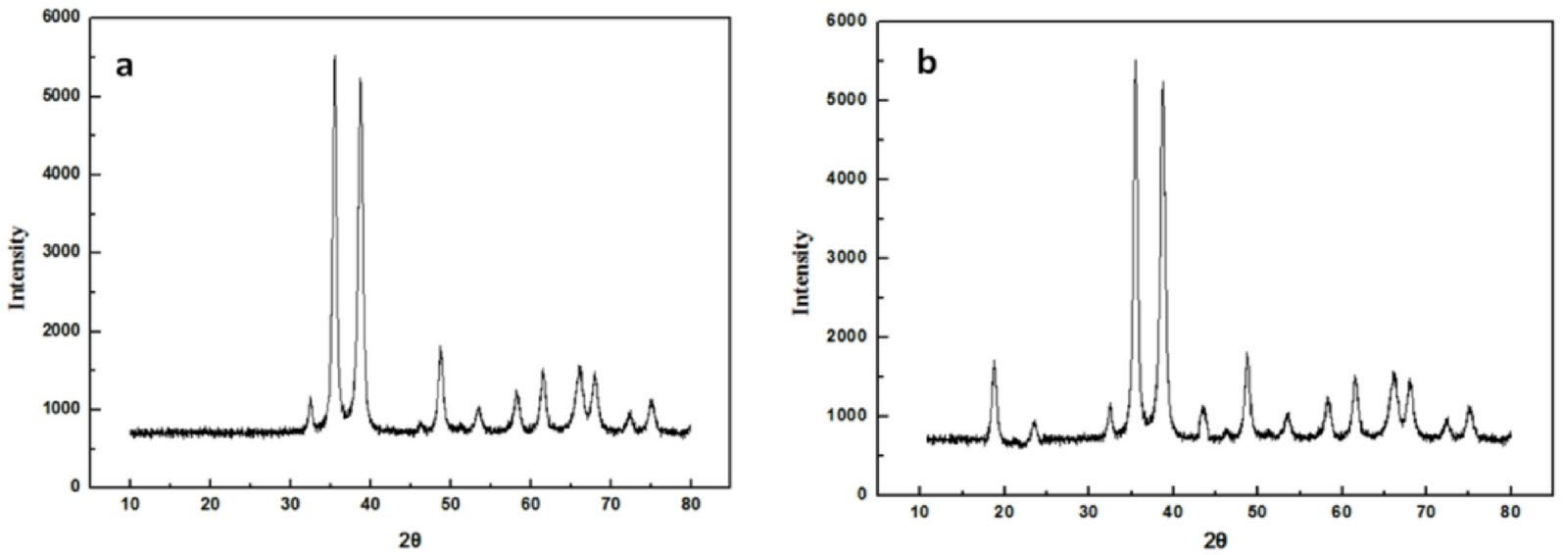
XRD images of (**a**) copper oxide nanowires and (**b**) ACF/CuO

Figure 4 presents the XRD patterns for copper oxide nanowires and ACF/CuO. Figure 4a depicts the XRD pattern of copper oxide nanowires, which was identified as pure CuO by comparison with the PDF card (05–0661). Figure 4b illustrates the XRD pattern of ACF/CuO. When compared to Fig. 4a, characteristic diffraction peaks of graphitization are observed at 2*θ*= 18.26° and 43.63°. This indicates that copper oxide crystals which are monoclinic CuO have been well-dispersed onto the surface of the activated carbon fibers [6, 7, 14].

### Removal of methyl orange in different reaction systems

**Fig. 5:**
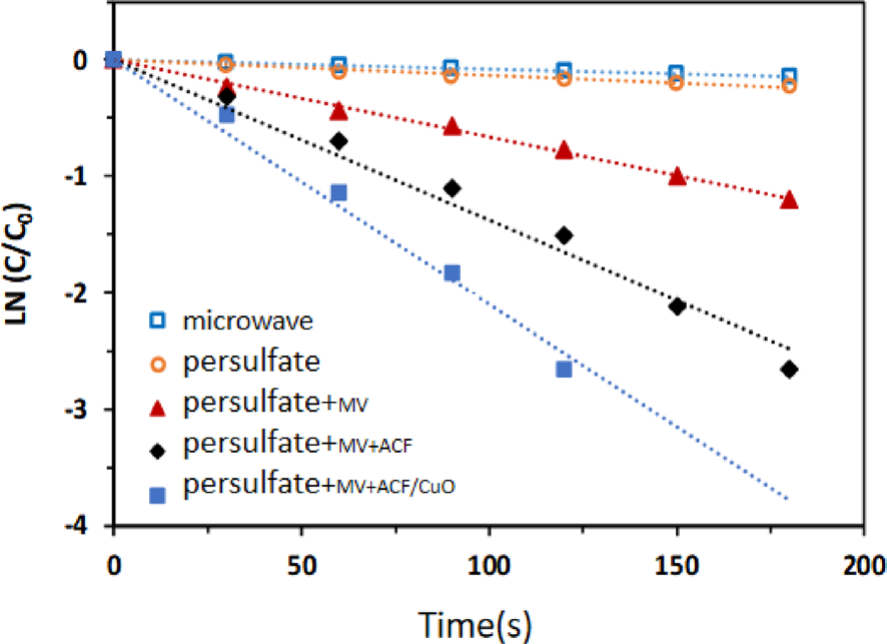
Removal of methyl orange in different reaction systems. Reaction conditions: microwave power was set at 500 W; irradiation time was 120 s; oxidant dosage was 0.6 g/L; methyl orange concentration was 100 mg/L

The microwave power was set at 500 W, the irradiation time was 120 s, the catalyst dosage was 10 g/L, the oxidant dosage was 0.6 g/L, and the pollutant concentration was 100 mg/L. The changes in methyl orange concentration within various reaction systems were examined, and the reaction rates were calculated. The experimental results are depicted in Fig. 5. As can be observed from the figure, when only microwave irradiation or potassium persulfate was present in the reaction system, the concentration of methyl orange remained essentially unchanged, and the reaction rates were also low, at (0.0008 ± 0.0001) and (0.0013 ± 0.0001) s^− 1^, respectively. In the combined microwave and potassium persulfate reaction system, the concentration of methyl orange decreased, and the reaction rate increased to (0.0064 ± 0.0001) s^− 1^. The reaction rate increased by approximately 8 times and 5 times when microwave irradiation was combined with potassium persulfate, compared to using microwave alone or potassium persulfate alone, respectively. This indicates that the microwave irradiation promotes the decomposition of potassium persulfate and the generation of free radicals for the degradation of organic matter. When activated carbon fiber was added to the microwave and potassium persulfate reaction system, the efficiency of the reaction system was further improved, and the reaction rate reached (0.0138 ± 0.0001) s^− 1^, which was approximately 2 times that of the microwave and potassium persulfate reaction system without the addition of activated carbon fiber. When ACF/CuO was used as the catalyst, the reaction rate of the system peaked at (0.0210 ± 0.0001) s^− 1^, which was approximately 1.5 times that of the microwave and potassium persulfate reaction system with the addition of activated carbon fiber. The above demonstration shows that activated carbon fiber loaded with CuO exhibits a higher activation activity for potassium persulfate than pure activated carbon fiber.

### Study on microwave-assisted K_2_ S_2_ O_8_ -ACF/CuO synergistic catalytic oxidation system and influencing factors

#### Effect of ACF/CuO dosage

**Fig. 6:**
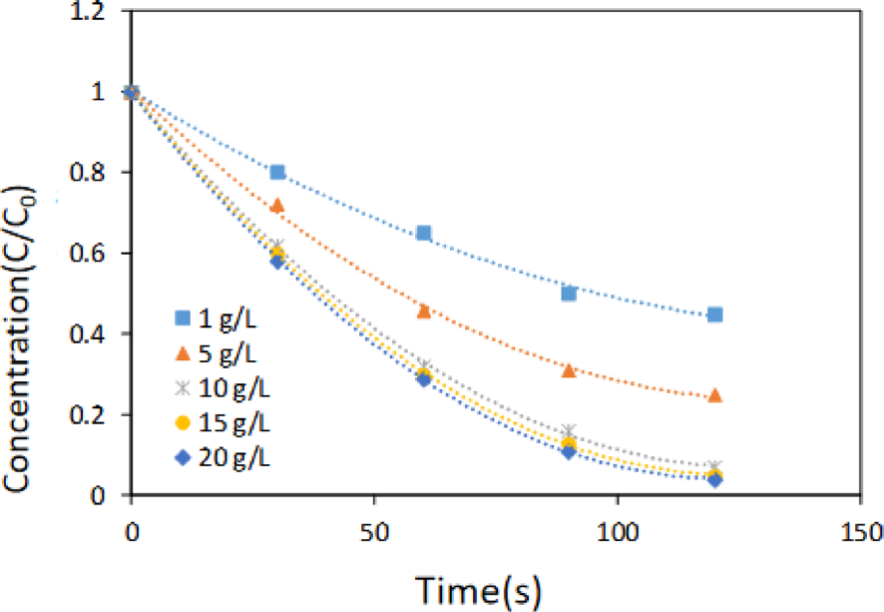
The effect of absorbance of methyl orange by dosage of ACF/CuO. Reaction conditions: microwave power was set at 500 W; irradiation time was 120 s; oxidant dosage was 0.6 g/L; methyl orange concentration was 100 mg/L

Adjust the microwave power to 500 W. Take fiber-activated carbon fiber loaded with copper oxide material at concentrations of 1 g/L, 5 g/L, 10 g/L, 15 g/L, and 20 g/L. Add 100 mL of a 100 mg/L methyl orange solution and potassium persulfate at a concentration of 0.6 g/L. Irradiate for 2 min, and the changes in methyl orange solution concentration are shown in Fig. 6.

As illustrated in Fig.6, the absorbance of the methyl orange solution gradually decreased with the progressive increase in the dosage of ACF/CuO. When the ACF/CuO dosage reached 10 g/L, the removal rate of methyl orange increased from (55 ± 1)% at 1 g/L to (93 ± 1)%. Upon further increasing the ACF/CuO dosage, the concentration of the methyl orange solution continued to decrease slowly. However, the removal rate of methyl orange remained essentially constant, which was due to reduced microwave penetration and overlapping energy absorption areas at high doses. Consequently, the optimal dosage of ACF/CuO was determined to be 10 g/L.

#### Effect of potassium persulfate dosage

**Fig. 7:**
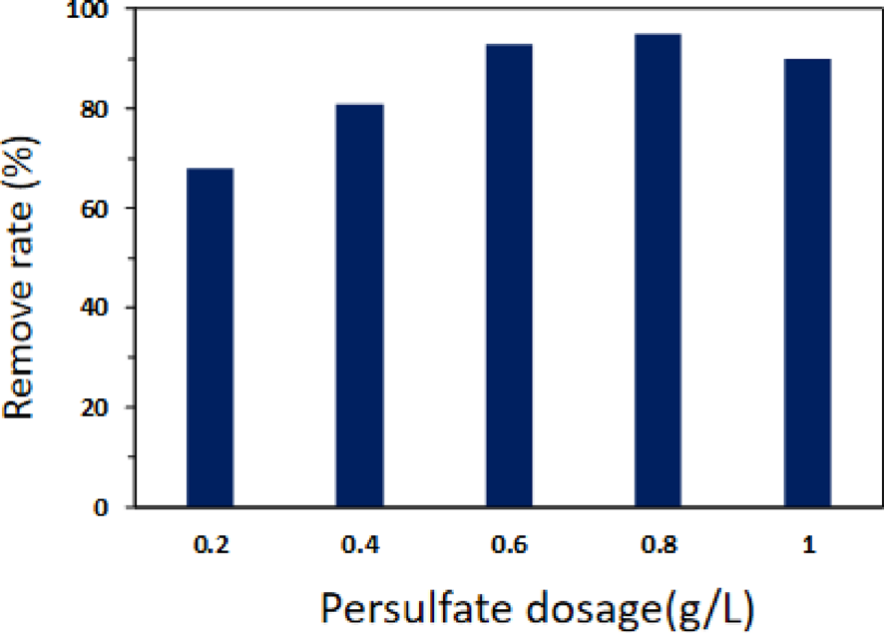
The effect of removal efficiency of methyl orange by dosage of K_2_S_2_O_8_. Reaction conditions: microwave power was set at 500 W; irradiation time was 120 s; ACF/CuO dosage was 10 g/L; methyl orange concentration was 100 mg/L

Adjust the microwave power to 500 W, add 10 g/L of fiber-activated carbon fiber loaded with copper oxide material, and 100 mL of a methyl orange solution with a concentration of 100 mg/L. Then, introduce potassium persulfate at concentrations of 0.2 g/L, 0.4 g/L, 0.6 g/L, 0.8 g/L, and 1 g/L. After irradiating for 3 min, observe the change in the reaction rate of the system, as depicted in Fig. 7.

As illustrated in Fig. 7 the removal rate of methyl orange in the microwave-potassium persulfate-ACF/CuO system progressively increased with the incremental addition of potassium persulfate. When the dosage was increased from 0.2 g/L to 0.4 g/L and then to 0.6 g/L the removal rate increased from (68.5 ± 0.2)% to (80.1 ± 0.3)% and then to (93.2 ± 0.3)%. At a dosage of 0.8 g/L the removal rate of methyl orange further increased to (95.4 ± 0.2)%. However when the potassium persulfate dosage was elevated to 1 g/L the removal rate unexpectedly dropped to 90%. This decline was attributed to the self-quenching reaction of excessive potassium persulfate which decreased the concentration of free radicals in the system thereby reducing the pollutant removal rate [15]. Consequently, the optimal dosage of potassium persulfate was determined to be 0.6 g/L.

#### Effect of microwave power

**Fig. 8:**
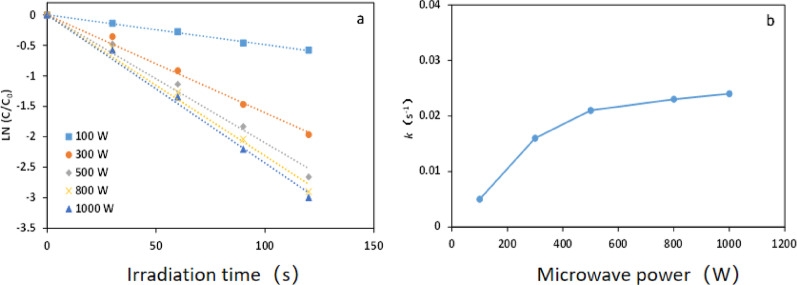
Effects of microwave power on reaction rate: **a** reaction ratio; **b** variation of *k* with microwave power. Reaction conditions: ACF/CuO dosage was 10 g/L; oxidant dosage was 0.6 g/L; methyl orange concentration was 100 mg/L

The microwave power was adjusted to 100 W, 300 W, 500 W, 800 W, and 1000 W. A sample of 10 g/L of fiber activated carbon fiber loaded with copper oxide was taken and added to 100 ml of a solution containing 100 mg/L of methyl orange. Potassium persulfate was dosed at 0.6 g/L, and the solution was then irradiated for 120 s. The absorbance change of the methyl orange solution was observed and is depicted in Fig. 8.

As illustrated in Fig. 8, the reaction rate of the microwave-ACF/CuO-potassium persulfate system progressively increased with the incremental rise in microwave power. When the microwave power reached 500 W, the reaction rate climbed from 0.005 at 100 W to 0.021 s^− 1^. However, upon increasing the microwave power from 500 W to 1000 W, the reaction rate only increased to 0.024 s^− 1^. Consequently, the optimal microwave power for this reaction system was determined to be 500 W.

#### Reutilization experiment of ACF/CuO

**Fig. 9:**
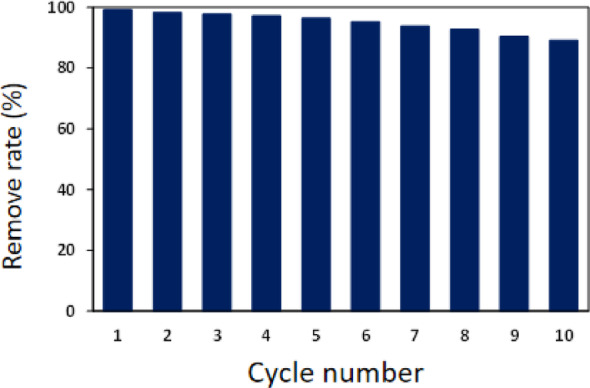
Recycling rate of ACF/CuO. Reaction conditions: microwave power was set at 500 W; irradiation time was 120 s; ACF/CuO dosage was 10 g/L; oxidant dosage was 0.6 g/L; methyl orange concentration was 100 mg/L. Method for the recovery of catalyst: filter paper filtration method

Set the microwave power to 500 W, the radiation time to 2 min, the potassium persulfate dosage to 0.6 g/L, and the ACF/CuO dosage to 10 g/L. The experiment was repeated 10 times under the same conditions. According to the changes in the decolorization rate of methyl orange, as shown in Fig. 9, it can be observed that with an increase in the number of tests, the removal rate of the methyl orange solution consistently reached 100% after ten reuses. This is because, during the reaction, ACF/CuO acts solely as a catalyst, utilizing its own hotspots to promote the production of SO_4_^−^ from persulfate without any consumption. Consequently, as a continuous catalyst in microwave advanced oxidation reactions, ACF/CuO not only reduces the energy consumption and cost of the reaction but is also reusable. The experiment confirms that ACF/CuO is an effective and environmentally friendly catalyst.

### Analysis of the mechanism in the microwave-potassium persulfate-ACF/CuO system

**Fig. 10:**
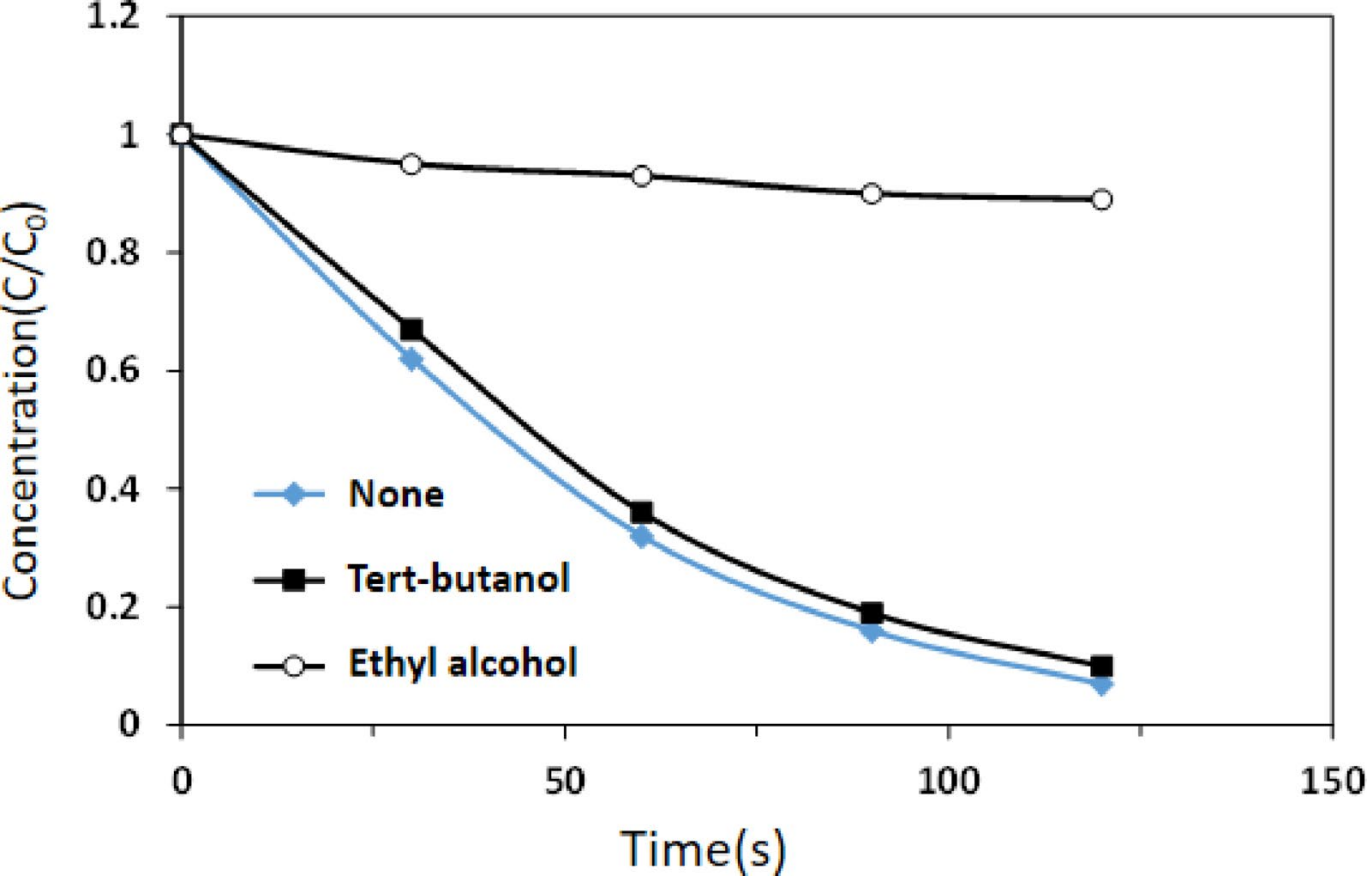
Effect of radical scavengers on the MO degradation. Reaction conditions: microwave power was set at 500 W; irradiation time was 120 s; ACF/CuO dosage was 10 g/L; methyl orange concentration was 100 mg/L

In the catalytic system, two types of free radicals are generated: sulfate radicals and hydroxyl radicals. These free radicals can efficiently degrade organic matter in water. Therefore, it is crucial to ascertain the role of each free radical within the catalytic system to explore the catalytic mechanism and subsequently achieve micro-control of the catalytic reaction. During the experiments, *tert*-butanol and ethanol were introduced into the reaction system under optimal conditions. The reaction rates of ethanol with sulfate and hydroxyl radicals were similar, whereas the reaction rate of *tert*-butanol with hydroxyl radicals was significantly higher than with sulfate radicals [3, 11, 21]. Consequently, by separately adding ethanol and *tert*-butanol to the reaction system, it was possible to determine which free radical played a more significant role in methyl orange degradation based on their impact on the degradation rate of methyl orange. The experimental results, depicted in Fig. 10, indicate that the addition of ethanol resulted in a dramatic decrease in methyl orange degradation efficiency. In contrast, the addition of *tert*-butanol did not lead to a significant change in the degradation of methyl orange compared to when no trapping agent was introduced.

The results above indicate that in the microwave-potassium persulfate-ACF/CuO system, sulfate radicals primarily drive the degradation process, while hydroxyl radicals contribute only minimally. Furthermore, it is evident that the free radicals produced within the system are predominantly sulfate radicals.

### Analysis of methyl orange degradation and transformation rules in microwave-potassium persulfate-ACF/CuO system

#### Analysis of methyl orange degradation process by high performance liquidchromatography-mass spectrometry

**Fig. 11:**
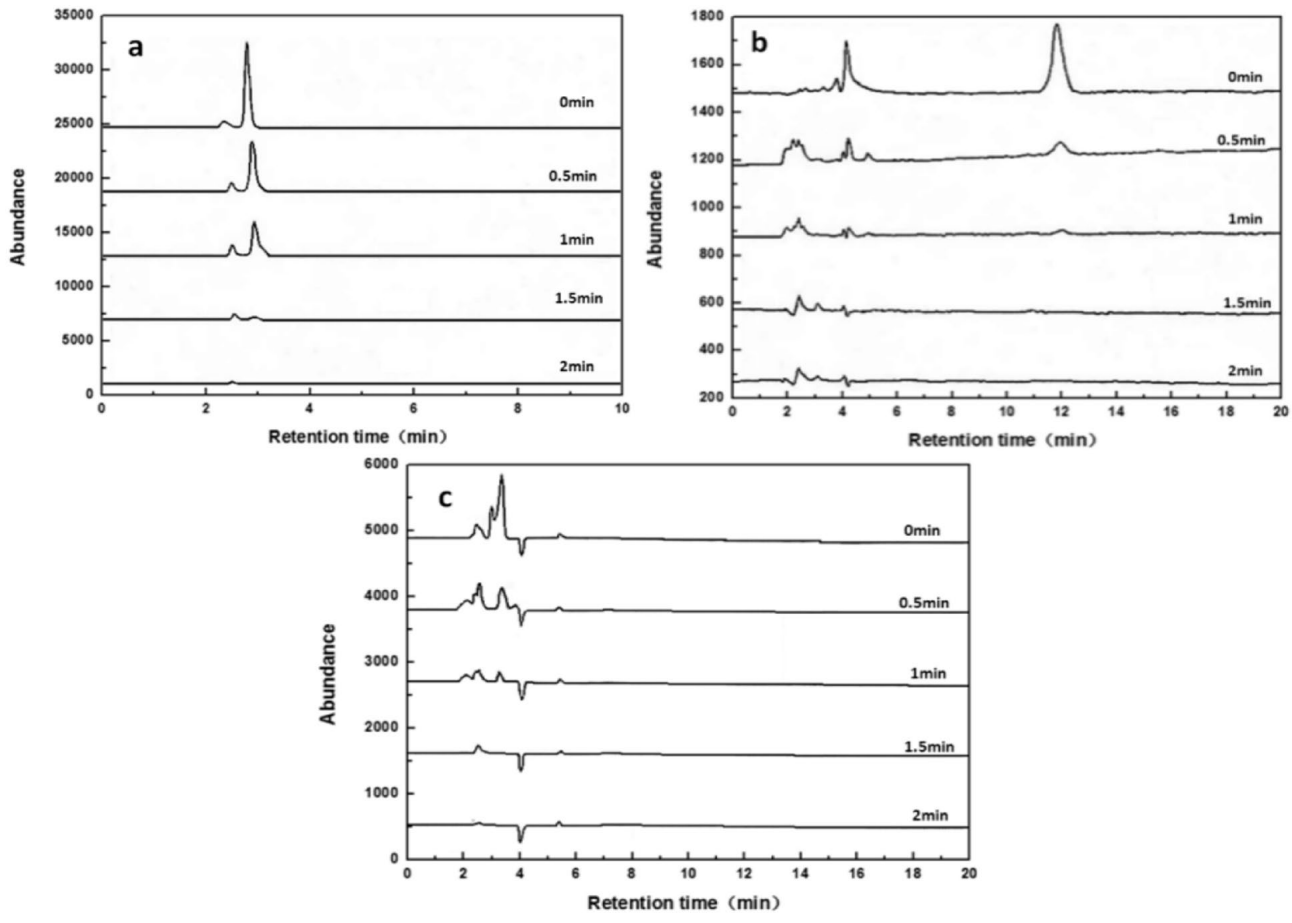
HPLC of methyl orange dye wastewater with various time by (**a**) 461 nm, **b** 283 nm, **c** 248 nm

Under optimal process conditions, samples of raw and treated water were collected, filtered, and diluted 50-fold. The treatment results of the methyl orange solution were analyzed via high-performance liquid chromatography, with an injection volume of 5 µL. The mobile phase had a V(methanol): V(water) ratio of 50:50, and the separation was carried out using a Shimadzu C18 column (4.6 × 150 mm) at a flow rate of 1.0 mL/min and a column temperature of 30 °C. The test results are shown in Fig. 11.

Figure 11 presents the high - performance liquid chromatograms of methyl orange aqueous samples at wavelengths of 461 nm, 283 nm, and 248 nm at various times. Figure 11(a) illustrates that at a wavelength of 461 nm, the retention time for methyl orange was 2.93 min, and the absorption peak of methyl orange diminished rapidly as time passed. This suggests that the chromophore group was damaged and methyl orange underwent degradation. Figure 11(b) indicates that for the organic compounds at 283 nm, with a retention time of 2.8 min, their quantity initially increased and then gradually decreased. This implies that organic compounds with a co-chromophoric structure were formed during the degradation of methyl orange, and these intermediates were progressively destroyed as the treatment time increased. Figure 11(c) reveals a new absorption peak at 248 nm, signifying that organic intermediates with a benzene ring structure emerged during the degradation process. As time went on, these intermediates were also degraded, leading to the complete breakdown of methyl orange.

#### Analysis of methyl orange degradation process

During the degradation of methyl orange, a series of intermediate products are produced. The generation of these intermediate products can effectively reveal the degradation process of methyl orange and can also reflect the reaction mechanism of microwave advanced catalytic oxidation technology in degrading methyl orange. Further analysis of the methyl orange solution treated by microwave advanced catalytic oxidation technology using GC-MS and comparison with the standard substances in the NIST spectral library revealed that the intermediate products were determined as shown in Table 1.

**Table 1:** GC-MS analysis of the intermediate of methyl orange

Time(min)	Name	CAS	Structural formula
10.32	benzenesulfonic acid	98-11-3	
16.63	*p*-nitrophenol	100-02-7	
18.08	maleic anhydride	108-31-6	

**Fig. 12:**
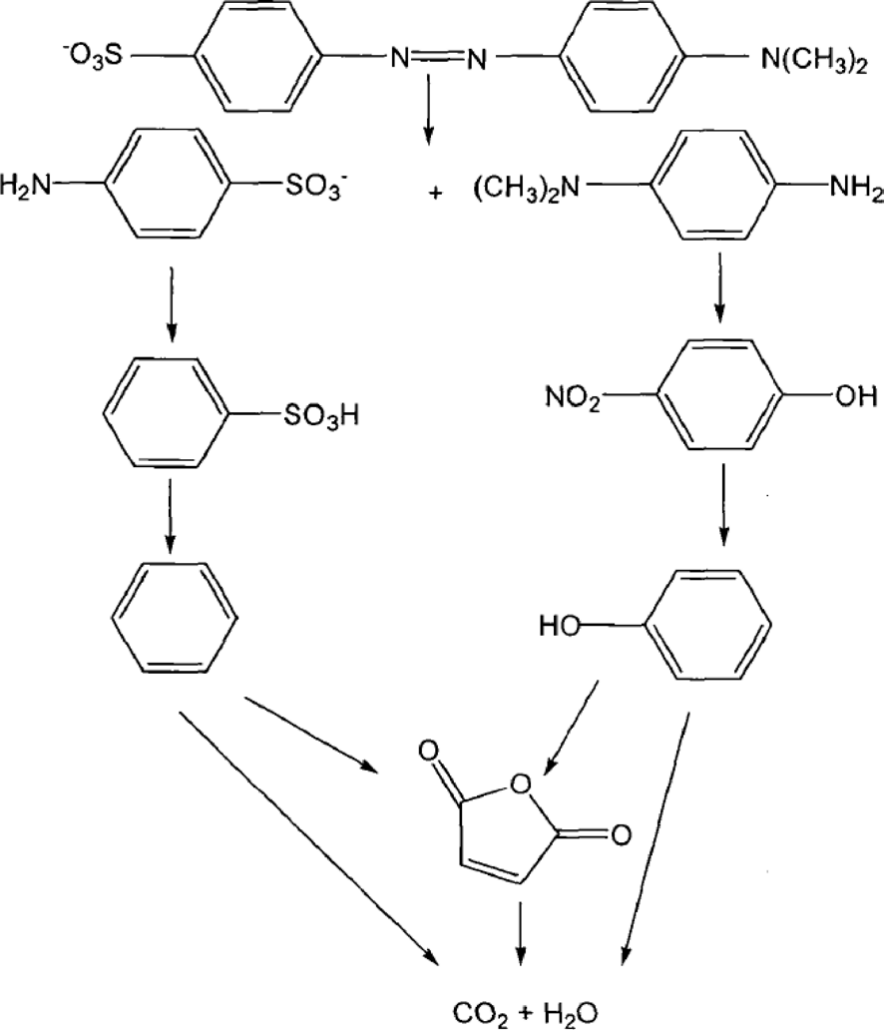
Degradation pathway of methyl orange

As indicated in Table 1, under the advanced catalytic oxidation of methyl orange by microwave, initially, the double bond is cleaved to produce benzenesulfonic acid and *p*-nitrophenol Hojjati-Najafabadi [4]; Maravilla Jr [12]. Subsequently, benzenesulfonic acid and*p*-nitrophenol undergo gradual degradation, transforming into a benzene ring and phenol. Through oxidation by sulfate radicals, portions of the benzene ring and phenol experience chain cleavage, resulting in the formation of maleic anhydride. Ultimately, both the benzene ring and phenol, along with the produced maleic anhydride, are degraded into water and carbon dioxide. Based on this analysis, the potential degradation pathways of methyl orange solution are depicted in Fig. 12.

#### Water quality analysis of methyl orange water samples before and after treatment

COD, TOC, and copper concentrations dissolved in the methyl orange solution were examined both before and after treatment, with the results presented in Table 2..

**Table 2.** Parameters of methyl orange dye wastewater by microwave-K_2_S_2_O_8_-ACF/CuO

	COD (mg/L)	TOC (mg/L)	Cs(Cu) (mg/L)
Before treatment	125.23	48.48	0
After treatment	13.34	13.40	5.00

As indicated in Table 2., the removal rates of COD and TOC before and after treatment were 89.65% and 72.36%, respectively. These results suggest that the microwave-potassium persulfate-ACF/CuO system effectively degrades the organic structure of methyl orange, rather than merely achieving decolorization. Additionally, the concentration of copper ions in the treated solution was 5.00 mg/L, indicating that the activated carbon fiber loaded with copper oxide can effectively address the issue of copper oxide dissolution. This is acceptable when the raw water is industrial wastewater.

##  Conclusion

1. The physical and chemical properties of the synthetic materials were analyzed by various detection methods. Scanning electron microscopy, Fourier-transform infrared spectroscopy, and X-ray diffraction (XRD) analyses confirmed the successful preparation of activated carbon fiber loaded with copper oxide. Scanning electron microscopy revealed that after pretreatment, immersion, and heat treatment, the smooth surface of the activated carbon fiber was uniformly coated with copper oxide fibers. Infrared spectroscopy and XRD results indicated the presence of both activated carbon fiber and copper oxide components.
2. The effects of multiple environmental factors on the experimental process were investigated. The dosage of ACF/CuO, potassium persulfate, microwave power, and radiation time all significantly influence the decolorization rate of methyl orange solutions. The optimal experimental conditions were determined to be a microwave power of 500 W, a radiation time of 2 min, a potassium persulfate dosage of 0.6 g/L, and an ACF/CuO dosage of 10 g/L, resulting in a decolorization rate of 93% for methyl orange solutions. Moreover, ACF/CuO retained a high decolorization rate even after being reused 10 times.
3. The reaction mechanism was analyzed in depth. Experiments involving the injection of free-radical trapping agents revealed that in the microwave-potassium persulfate-ACF/CuO system, sulfate radicals are primarily responsible for the degradation process, and there is minimal generation of hydroxyl radicals during the catalytic reaction.
4. The potential degradation mechanism of methyl orange under microwave advanced catalytic oxidation was elucidated through liquid chromatography and gas chromatography - mass spectrometry (GC-MS) analyses. The testing of actual water samples indicated that the removal rates for chemical oxygen demand (COD) and total organic carbon (TOC) were 89.65% and 72.36%, respectively. Moreover, the concentration of copper ions in the treated solution was 5.00 mg/L, which implies that activated carbon fiber loaded with copper oxide effectively resolves the problem of copper oxide dissolution.

## Data Availability

The datasets used and/or analysed during the current study are available from the corresponding author on reasonable request.
